# Assessing and Enhancing Health Care Providers' Response to Domestic Violence

**DOI:** 10.1155/2014/759682

**Published:** 2014-04-22

**Authors:** Tuija Leppäkoski, Aune Flinck, Eija Paavilainen

**Affiliations:** ^1^School of Health Sciences, (Nursing Science), University of Tampere, Finland; ^2^The District Hospital of Southern Ostrobothnia, Finland; ^3^National Institute for Health and Welfare, Finland

## Abstract

This study aimed to examine possible changes from 2008 to 2012 in the skills of health care staff in identifying and intervening in domestic violence (DV). A longitudinal descriptive study design with volunteer samples (baseline; *n* = 68, follow-up; *n* = 100) was used to acquire information regarding the present state and needs of the staff in practices related to DV. The results of the baseline survey were used as a basis for planning two interventions: staff training and drafting practical guidelines. Information was collected by questionnaires from nurses, physicians, and social workers and supplemented by responses from the interviews. The data were analysed using both quantitative and qualitative methods. A chi-square test was used to test the statistical significance of the data sets. In addition, participants' quotes are used to describe specific phenomena or issues. The comparison showed that overall a small positive change had taken place between the study periods. However, the participants were aware of their own shortcomings in identifying and intervening in DV. Changes happen slowly, and administrative support is needed to sustain such changes. Therefore, this paper offers recommendations to improve health care providers' response to DV. Moreover, there is a great need for evaluating the training programme used.

## 1. Introduction

Domestic violence (DV) is globally recognised as a major but underreported public health and social problem among heterosexual and same-sex couples [1, 2]. It results in injuries and other negative short- and long-term effects on the health of all the family members [3, 4]. Children and young people in families where DV has taken place are at risk of abuse and associated detrimental health outcomes [5–7]. Nurses and physicians play a vital role in addressing these problems. Early identification of DV can reduce its consequences and may help to prevent further violence.

Unfortunately, health care professionals do not engage with these issues and they do not routinely screen for health risks such as DV or child abuse (CA) and neglect. In a Swedish study by Sundborg et al., only half of the nurses working in primary health care always asked women about DV and did so mostly when the patient was physically injured [8]. Health professionals do not ask about or identify DV, even in cases where it is obvious [9–12]. According to a Finnish study by Husso et al., it seems that there is a tendency for health care staff to focus on fixing the injuries and consequences of DV while dismissing the violence that is the cause of symptoms and injuries [13]. Consequently, asking about violence is undesirable. On the other hand, patients generally find being asked about violence acceptable [7, 8, 11, 12].

Various studies have shown that nurses and doctors ascribe their reluctance to or discomfort with inquiring about DV to factors such as lack of time, behaviours attributed to women living with abuse (e.g., denial), lack of training and effective interventions, the complexities of providing whole family care, and partner presence [10, 11, 14–16]. A lack of knowledge of the causes and effects of DV often leads to feelings of inadequacy and frustration [13]. For example, trauma caused by DV is not always recognized by health care professionals. The victim's trauma may affect the victim's ability to discuss the problem. Furthermore, nurses who work in a fast-paced environment may be used to seeing immediate results when they intervene with patients, whereas DV is a complex issue and not the one that can be solved in one visit [17]. Overall, awareness of one's own attitudes, myths, and stereotypes (e.g., men are always offenders; violence is only physical; violence only concerns marginal groups) plays an important role in one's readiness to deal with intimate partner violence (IPV) [8, 11, 17, 18].

The most frequently reported facilitators to ask about DV, alongside training community resources and professional tools, are protocols and police [7, 15]. Multifaceted and intersectoral approaches that address the individual, interpersonal, workplace, and systemic issues faced by nurses and physicians when inquiring about DV are required [15]. It has also been mentioned that strong leadership and prioritization of the issue have facilitated the development of the care process to detect and manage DV presentations [19]. Training and organizational change within healthcare systems can increase the identification and knowledge of DV, as well as health professionals' readiness to ask victims about it [16, 19–21]. However, so far, the effect of systematic screening for DV has remained somewhat unclear. Randomized controlled trials have shown that there is insufficient evidence to recommend routine screening for DV [22, 23].

The Finnish national publication “Recommendations for the prevention of interpersonal and domestic violence” (2008) stresses local and regional work and the importance of strategic planning, in addition to training [24]. A “National clinical nursing guideline for identifying and intervening in child maltreatment within the family in Finland” has also been drawn up based on practical work [25].

During this research project, the staff participating in the study was trained to recognise and address DV. Finally, the follow-up survey was conducted. The study searched for answers to the following questions. (1) How did the staff's ability to detect violence change during the research period of 2008–2013? (2) How did the staff's readiness to intervene in violence change during this time period?

## 2. Participants and Methods

### 2.1. Participants

The study began in 2008 with an initial survey of health care professionals in a large central hospital and in one local primary health care organization. The participating units were an emergency clinic and a doctors' office in primary health care, an emergency clinic, an orthopaedic ward, and an acute psychiatry emergency unit in specialized health care. The staff profile of the study included physicians, nurses, and social workers. The idea was to enhance not only the knowledge, skills, or attitudes of individuals with respect to domestic violence but also interdisciplinary understanding and collaboration.

### 2.2. Study Design

This is a longitudinal study with a pre-/posttest design. An initial survey was used to gain information of the present state and needs of the health care workers in primary and specialized health care with respect to DV.

The results of the survey were used as a basis for planning follow-up interventions: staff training and drafting practical guidelines.

The questionnaire asked for the following information: the participants' demographic data (gender, age, occupation, length of time in current occupation, and employment status), the prevalence and/or treatment of DV (e.g., “Can you estimate how often you meet or treat women or men who are victims of DV?” “Can you estimate how often you meet or treat women or men at work who are perpetrators of DV?” “At work, have you met or treated men who have experienced DV?”), the identification of and intervention in DV (e.g., “Do you believe you would identify a patient who is experiencing or has experienced DV?” “Do you believe you would identify a patient who is or who has been violent in their relationship?” “Is there an operations model in your work unit for intervening in DV?” “Do you collaborate with different support authorities when meeting the victim and the perpetrator?” Furthermore, the participants were asked to identify issues that may be a barrier to the recognition of DV and the actions of the health care personnel with a patient who has experienced violence or who has used violence.), the quality of the DV training received (e.g., “Has DV been discussed in your professional basic training?” “Have you participated in training organized by your employer?”), and one open-ended question comprising the DV work in the participant's unit within the last two years (see Tables 1 and 2).

In addition, qualitative data were gathered by interviewing health care professionals, during two group interviews, police, a social worker, and crisis workers about their experiences regarding cooperation in practice, including barriers and possibilities, shared responsibilities, and motivation to react to DV.

The outcome evaluation data were collected in May 2012 with the same instrument as at the beginning of the project, in 2008. In addition, qualitative data were gathered by interviewing health care professionals (*n* = 6).

### 2.3. Interventions

The educational intervention was planned on the basis of the results of the initial survey [26] and research evidence from earlier studies (e.g., [9, 27, 28]) and was completed over a four-month period, from January 2008 to May 2008. The training was carried out over three training sessions. The sessions were repeated twice with the same content so that as many shift workers as possible could participate. The themes of the sessions were orientation (2 × 4 h), DV from an ethical and legal point of view and raising the problem in discussion (2 × 7 h), and local, regional, and national service networks in DV and solving problems.

At the same time of the training sessions, a development process was started to create practical guidelines to be used as a tool for determining how to identify, respond, and intervene in the following situations: where there are more reasons to suspect DV, when DV is brought up by the patient, and when the symptoms and signs of the happened DV are noticeable. The idea was that the staff can immediately apply the knowledge gained from the course into practice. A multidisciplinary team that comprised staff nurses, social workers, and physicians worked together with the researchers. Over the years 2011–2013, the devised guidelines [29] have been integrated and implemented in practice to help and encourage the health care staff to identify and intervene in DV. So far, a total of 14 information events have been held with 237 participants. In addition, a project worker has visited different units to talk about the issue.

### 2.4. Data Analysis

Frequency tables were used to examine all variables. Pearson's chi-square test was used to test the changes in opinions between 2008 and 2012. Statistical significance was set at the level of 5% (*P* < 0.05). Because of the low number of answers to some questions, the data were combined into two groups.

Direct quotes were used to describe the participants' experiences and attitudes regarding the DV interventions that they had made.

### 2.5. Ethical Issues

Ethical approval (R12857H) for the project was granted by the Ethical Committee of Pirkanmaa Hospital District. No personal data of the staff were recorded. Quotes have been used in such a way that the informants are not recognizable.

## 3. Results

### 3.1. The Participants' Demographic Data

The 2008 sample consists of 68 respondents and the response rate was 35%. The 2012 sample consists of 100 respondents and the response rate was 50%. There have been no significant differences in the respondent demographics (gender distribution, occupational status, length of time in current occupation, and full- or part-time employment) during the study years of 2008 and 2012. In both years, less than one-sixth of the respondents were men. Ninety percent of the respondents are nurses with varying levels of education and 10% are other personnel, such as social workers and doctors (Table 1).

In contrast, the age of the personnel differs to some extent between the study years. In the 2008 data, the median age of the respondents is 45 years. In the 2012 data, the median age of the respondents is 43 years. However, the difference is not significant. In the 2008 data, respondents between 40 and 49 years formed 37% of all respondents, whereas, in the data from 2012, they form less than a quarter of all the respondents (Table 1).

### 3.2. Identification of a Victim of DV and a Patient Who Has Used Violence

The results of the initial survey (26) revealed that the staff had different kinds of barriers to identification of and intervention in DV, for example, a lack of mentoring and role modelling and a perceived lack of privacy and time available. Furthermore, there were no commonly agreed multiprofessional practices to identify and help the parties of DV. By the results of the initial survey the training was seen as incidental and the staff needed it.

In both study years, nearly all respondents (>90%) had met women who had experienced violence. The participants reported that they had met more men who had experienced violence in 2012 when compared to the 2008 data (40% versus 60%; *P* = 0.009). According to the surveys, approximately one-third of the respondents reported that at least once a month they met or treated women and men who had experienced DV. The participants also reported roughly equal numbers of both male and female perpetrators of violence during both study years. Approximately one-fourth of the respondents reported that they meet perpetrators of violence at least once a month (Table 2).

In the 2012 study, well over half of the respondents believed that they always or mostly identify a patient who experiences and/or has experienced DV. This is approximately 10% more than in 2008, but the difference is not statistically significant. On the other hand, the number of respondents who reported that they are able to identify a patient who has used violence in their intimate partnership increased from less than one-fifth in 2008 to over two-thirds in 2012 (*P* = 0.040) (Table 2).

The participants were asked to name 1–6 things that raise the suspicion of violence, even if the patient did not mention it. In both study years, the most suspicious points were the patient's physical injuries (e.g., typical injuries such as bruises around the body or the nature of the injuries not matching what the patient reports that has happened) and the patient's behaviour (e.g., the patient cannot give an explanation for the injuries; the patient cannot say where the injuries originate; the patient declines follow-up treatment; first the patient admits and then denies violence). Both points cover altogether approximately 60% of all mentions of points that raise suspicion. The remaining 40% were related to vague symptoms reported by the patient (e.g., stomach pain, chest pain, and a headache) without a corresponding cause, the worker's intuition becoming aroused, and the behaviour of the perpetrator of violence if they are accompanying the patient. In both study years, >80% thought that identification of violence is difficult (very or fairly difficult) (Table 2). In both study years, nearly four-fifths of the respondents (77%) thought that their readiness to identify DV was moderate.

The participants were asked to identify issues that may be a barrier to the recognition of DV. It was possible to circle several given alternatives (1–13). The largest group consists of the following: “the patients are reluctant to say that DV happened” and “when asked, a patient exposed to DV does not admit it” (two-thirds of all alternatives). Matters related to the identification of acute violence or symptoms, a lack of time to ask about violence, or the work environment lacks a peaceful place where to ask violence were mentioned the least.

Furthermore, the respondents were asked to name things that they think would prevent intervention in DV. In this task, the respondents had the possibility to check all suitable options, with altogether 16 options given in the survey form. In both study years, the protection of the perpetrator of violence, the patient not admitting to having experienced violence when asked, and the patients being unwilling to discuss the matter if the perpetrator is present during the appointment were mentioned the most. These points covered 60% of all responses. The lack of time or space was not an obstacle to intervention in violence and very few nurses felt that they would be interfering in the patient's personal matters when asking about violence.

In 2008, apart from a couple of respondents, 97% of the respondents thought that intervention in DV is difficult (very or fairly difficult). According to the 2012 survey, 90% thought that intervention is difficult and 10% of the respondents thought that it was easy (Table 2).

The participants explained the difficulty as being caused by the patient denying the violence, the phenomenon of violence being a sensitive issue, and the patient reporting the reason for coming to health care to be something other than the violence they have experienced. The comment that “intervention in violence is not seen as important” made by one respondent reflects the attitude of how to deal with DV. In both study years, approximately two-thirds of the respondents (70%) thought that their readiness to intervene in DV was at least moderate.

The demographic factors presented in Table 1 (gender, age, full- or part-time employment, and duration of working in the profession) were not observed to have a connection between the identification of and intervention in violence.

### 3.3. The Actions of the Health Care Personnel with a Patient Who Has Experienced Violence or Who Has Used Violence

A multiple-choice question was used to inquire about the actions of the health care personnel with a patient who is a victim of violence. This question offered the possibility to choose all suitable options (1–12). In both study years, the following actions were mentioned the most: “I ask the patient directly,” “I encourage my patient to report the offence if it is physical violence," and “I discuss the issue with the patient and follow up on the matter, with the patient's permission, by contacting supporting parties.”

Furthermore, the survey form inquired about the participants' actions with a patient who has used violence. This question also included the possibility to check all suitable options (1–10). In both study years, approximately one-fourth of the respondents' opinions are concerned with the personnel's intercommunication and how to act with a perpetrator of violence.

A statistically significant difference was seen between the respondent groups of the two study years when inquiring about whether the respondent's work unit had an operations model or practical guidelines for intervention in DV. In 2008, 51% of the respondents thought that there was no such model. For 2012, the equivalent figure was 12.5% (*P* < 0.000). However, in 2012, approximately half of the respondents could not say whether the work unit had an operations model or not (Table 2).

The respondents were also asked about the practice in their work unit of asking their clients systematically about DV. In 2008, two-thirds of the respondents thought that DV was not asked about systematically and 23% could not say whether it was systematically asked about or not. For 2012, the equivalent figures were 63% and 33%, but the difference is not statistically significant.

### 3.4. Service Networks and Multiprofessional Activity

There was no change over time with respect to collaboration between local supporting authorities. In both respondent groups, approximately half of the respondents reported that they collaborate with local supporting authorities (Table 2). The respondents were asked to describe the central collaborative parties and practices. In both study years, the respondents mentioned the police, social services, and crisis and emergency services as the closest collaborative parties. The nature of the contact with the collaborative parties was described in fairly general terms, such as a phone call, contact with the aforementioned supporting parties, or giving information to the patient about the different supporting parties. A couple of respondents described the matter in more detail: “I mostly ask about shelters,” “I contact the social emergency services if there are children at home,” or “I follow up on the matter with the patient's permission by contacting the police, for instance.”

### 3.5. Training

A statistically significant difference was found between the respondent groups for the question that asked whether DV has been discussed in professional basic training. In 2008, less than one-fourth of the respondents reported that their training had discussed DV. According to the 2012 survey, 45% of the respondents thought that matters related to violence had been discussed in professional training (*P* = 0.006). A statistically significant difference between the respondent groups was also found regarding how aware the respondents were of training organized by their employer. In 2008, approximately one-sixth of the respondents thought that their employer had organized training related to DV, and, according to the second survey, approximately half of the respondents were aware of training organized by their employer (*P* < 0.000). Moreover, 57% of the respondents had also participated in the training (Table 2). Participation in training organized by some other parties was marginal. In both study years, only about 8% of the respondents reported having participated in such training.

In both study years, training needs were concerned with the identification of and intervention in DV and helping both victims and perpetrators of DV. Matters of legislation in situations of DV were the least interesting topics of training. In both study years, a couple of respondents (2%) thought that they did not need any training.

Lastly, one-fourth of the respondents describe with a couple of words the development work related to violence in their own unit. Positive things were related to an awareness of training having been organised and the existence of written guidelines on how to act in DV situations. According to a couple of respondents, “the matter is discussed quite well if there is a suspicion of violence,” “in acute situations we aim to find out the patient's situation holistically,” and “the issue of violence is discussed more often these days.” The respondents mentioned the following as negative aspects: “the development work did not affect practical work in any way and the whole thing remained theoretical,” “the developed model does not work in practice,” “they could not fit all the employees in the training who wanted to attend,” and “there has only been training for a couple of employees.” A couple of respondents had recently started work at the unit and were not aware of the existence of the written guidelines.

## 4. Discussion

In both study years, over 90% of all respondents had met and treated women who had experienced DV. However, a difference was seen in questions regarding the recognition and treating of men who have experienced violence. In 2012, the respondents reported to have met or treated approximately 20% more men who have experienced DV than in 2008. This kind of study result can be a sign of the training having had an impact on the sensitivity of the participants towards identifying these men, even though a statistical difference was not seen between the training and the meeting of male victims of DV. According to a national report by Heiskanen and Ruuskanen [30], equal numbers of women and men experienced victims of partner abuse and over half of the men have had physical or psychological consequences from the violence. In addition, men may have more recurring experiences of violence [30]. As the consequences of violence are far reaching and complex, it is likely that these men seek treatment also in the units participating in the ongoing research project, and awareness of this fact could help practitioners to identify and treat them.

The identification of and intervention in DV were still considered difficult in the survey of 2012, and the training received was not observed to have a significant connection to these matters. Moreover, no significant changes have happened in the actions of the health care personnel with victims and perpetrators of violence between 2008 and 2012. Over the four years, there were no differences in the number of interventions to help victims and perpetrators of violence. In 2012, slightly more interventions were made with patients who have perpetrated violence. The respondents also thought that they identify patients who have used violence more often than those in 2008. The lack of time or space was not an obstacle to intervention in violence. This is an opposite result compared to the previous studies [7, 8, 10, 14–16]. Instead, the participants explained the difficulty as being caused by the patient denying the violence and the phenomenon of violence being a sensitive issue. This result is parallel to the earlier studies [7, 9, 11].

The participants acknowledged their weaknesses, as they named identification and intervention as the most important topics of supplementary training. A systematic further training plan for all staff should be developed in order to enhance professionals' skill and knowledge of all types of DV and how to identify and intervene in DV and CA. Organisational support, for example, guidelines or collaboration with others, has been mentioned as a shortcoming in earlier studies [8, 15]. Some nurses were unaware of the written guidelines and the legal points of views. For some respondents, the operations model developed had remained theoretical and they thought that it did not translate to practical work. “The matter is easily filed away and forgotten,” as one employee noted, but they also added that “it is also an issue of work management.”

McCloskey et al. have concluded that the content of the training received was more important than the amount of training received [31]. In the future, it is important to emphasize more the use of interactive methods, such as role playing, and to involve the supporting parties (the supporting network) in the training programmes. Further, the earlier results revealed that the staff lacked mentoring and role modelling [15, 24]. Thus, a mentoring action plan might help the health care staff with DV work.

Although more than one-third of the respondents in the second survey thought that the operations model for intervention in violence existed in their work unit, over half of the respondents still did not know whether one existed or not. This kind of study result may be affected by the fact that the survey was conducted approximately one year after the operations guideline had been adopted and orientation was still unfinished in the units. Whatever the reason is, the fact remains that not all respondents had been familiarized with their use or even their existence. The study result supports the idea that it is necessary to conduct a third survey in 2014 before ending the project. There have also been organizational changes during the study period and operations and functions have been reorganized. Due to this, there have been personnel transfers between units. Furthermore, in the 2008 data, nearly one-third of the respondents were temporary replacement workers, and, in 2012, nearly one-fourth of the respondents were temporary replacement workers. This kind of study result emphasizes the fact that supplementary training must be systematic and continuous. Moreover, the operation model or practical guidelines will also be a tool for the orientation of new staff.

According to this study, the units do not systematically ask about violence. This study result is consistentwith recent international research results [8, 11, 18]. In 2008, less than one-fourth of the respondents and one-third in 2012 did not know whether violence is asked about in their unit or not. At the moment, the guidelines do not include the idea of adopting a screening tool, but the issue is worth considering in the near future. Further, it lacks evidence whether DV screening reduces violence or improves health outcomes for victims [22, 23]. This demands further research.

Like earlier studies have stated, collegial and organisational support are needed [8, 15, 19]. Perhaps health care staff still lacks real collegial and organizational support, though in this case it would appear to be due to individual differences between units. One interviewee stated that “we have been supported by employees and our manager and we have made a violence folder with my work partner,” while another one stated that “the unit management is not committed to the matter.” Furthermore, it emerged from the open responses that only some of the personnel were able to participate in the training programmes. This means that, in the future, resources have to be allocated to supplementary training. Managers should take care of the resource allocation as well as making sure that violence training is included in annual training plans.

*Limitations.* Some limitations of the present study must be considered. First, these include the small sample sizes in both survey years. Second, the instrument used is based on the participants' report data. Third, the guidance implementation took place in 2011, one year before the follow-up survey. Fourth, perhaps those who participated in the surveys are individuals who are willing to take part in work against violence, thus creating selection bias. We noted that many familiar people took part in both training sessions and many other activities during the study period. Some of them worked as contact persons for their units. Furthermore, the respondents of the 2012 survey may not necessarily be the same as those in the 2008 survey, due to part of the personnel being temporary workers and some of them may have changed to a different workstation. Thus, it is a little bit difficult to clarify the degree of congruence between the reported data and the actual situation of identifying and intervening in DV. Because the research results might be biased, it is a little bit difficult to clarify whether the change was real or not.

## 5. Conclusions

Future research needs to evaluate our training programme as a process (process evaluation) and go through all the written feedback received. In this case, we will pay attention to, among other things, training activities or practical support, to find out if it is being delivered as planned and to identify gaps between its intended and actual deliveries. After that we must change the key points in the training programme. Further, we must cooperate with the nursing managers of the staff so that continuing education on DV issues is included in the annual training plans. We also need to continue to orient and familiarize the practitioners with using the guidelines because some of the respondents thought that the guidelines, written in collaboration, had remained theoretical. It is also necessary to consider other kinds of support tools such as screening tools. Naturally, the efficiency of these screening tools should also be evaluated and their adoption requires orientation of the practitioners. Because the implementation of the guidelines will take time, a new survey should be conducted in a couple of years in order to see development over a longer time period.

## Figures and Tables

**Table 1:** Demographic data on participants.

Variable	Category	Initial survey 2008 (*n* = 68)	Follow-up survey 2012 (*n* = 100)
Gender	Male	15%	14%
	Female	85%	86%

Age (years)	Mean	42	41
	Median	45	43
	Range	(24–59)	(21–60)
	Standard deviation	10	12

Age group	≤29	16%	29%
	30–39	23%	14%
	40–49	37%	23%
	50–59	24%	32%
	≥60		2%

Occupational status	Nurse	65%	62%
	Practical nurse	25%	28%
	Social worker	4%	
	Doctor	6%	2%
	Others		8%

Occupation time	Mean	16	16
	Median	15	15
	Range	(0.5–37)	(0.3–40)
	Standard deviation	11	12

Employment	Permanent	71%	76%
	Temporary	29%	24%

**Table 2:** Changes of opinions of the respondent groups to manage DV.

Questions		Initial survey 2008	Follow-up survey 2012	Significance test		
				*χ* ^ 2^	df	*P* value
Can you estimate how often at work you meet or treat women or men who are victims of DV?	At least once a month	(22) 33%	(28) 29%			0.558
	Once a month or less	(45) 67%	(70) 71%			

Can you estimate how often at work you meet or treat women or men who are perpetrators of DV?	At least once a month	(15) 23%	(25) 26%			0.684
	Once a month or less	(51) 77%	(73) 74%			

At work, have you met or treated men who have experienced DV?	No	(40) 60%	(39) 39%	6.897	1	**0.009**
	Yes	(27) 40%	(61) 61%			

Do you believe you would identify a patient who is experiencing or has experienced DV?	Always or often	(29) 43%	(54) 55%			0.155
	Once or never	(38) 57%	(45) 45%			

Do you believe you would identify a patient who is or who has been violent in their relationship?	Always or often	(13) 19%	(34) 34%	4.227	1	**0.040**
	Once or never	(54) 81%	(66) 66%			

Do you think that identification of DV is difficult or easy?	Difficult	(60) 88%	(82) 86%			0.747
	Easy	(8) 12%	(13) 14%			

Do you think that intervention in DV is difficult or easy?	Difficult	(63) 97%	(86) 90%			0.475
	Easy	(2) 3%	(10) 10%			

Is there an operations model in your work unit for intervening in DV?	No	(34) 51%	(12) 13%	35.761	2	**<0.000**
	Yes	(4) 6%	(34) 35%			
	Cannot say	(29) 43%	(50) 52%			

Do you collaborate with different supporting authorities when meeting the victim and the perpetrator?	No	(32) 51%	(47) 49%			0.821
	Yes	(31) 49%	(49) 51%			

Has DV been discussed in your professional basic training?	No	(49) 77%	(53) 55%	7.577	1	**0.006**
	Yes	(15) 23%	(43) 45%			

Has your current employer organized supplementary training related to DV?	No	(37) 56%	(29) 30%	22.585	2	**<0.000**
	Yes	(10) 15%	(50) 52%			
	Cannot say	(19) 29%	(18) 18%			

Have you participated in training organized by your employer?	No	(60) 92%	(54) 57%	23.014	1	**<0.000**
	Yes	(5) 8%	(40) 43%			

## References

[1] Krug EG, Mercy JA, Dahlberg LL (2002). *The World Report on Violence and Health*.

[2] Baker NL, Buick JR, Kim SR (2013). Lessons from examining same-sex intimate partner violence. *Sex Roles*.

[3] Johnson MP, Ferraro KJ (2000). Research on domestic violence in the 1990s: making distinctions. *Journal of Marriage and Family*.

[4] Campbell JC (2002). Health consequences of intimate partner violence. *The Lancet*.

[5] Holt S, Buckley H, Whelan S (2008). The impact of exposure to domestic violence on children and young people: a review of the literature. *Child Abuse and Neglect*.

[6] Nicklas E, Mackenzie MJ (2013). Intimate partner violence and risk for child neglect during early childhood in a community sample of fragile families. *Journal of Family Violence*.

[7] Olive P (2007). Care for emergency department patients who have experienced domestic violence: a review of the evidence base. *Journal of Clinical Nursing*.

[8] Sundborg EM, Saleh-Stattin N, Wändell P, Törnkvist L (2012). Nurses’ preparedness to care for women exposed to Intimate Partner Violence: a quantitative study in primary health care. *BMC Nursing*.

[9] Leppäkoski T (2007). *Women exposed to acute physical intimate partner violence seeking care at emergency departments—Identification of and intervention in violence [Ph.D. thesis]*.

[10] Robinson L, Spilsbury K (2008). Systematic review of the perceptions and experiences of accessing health services by adult victims of domestic violence. *Health and Social Care in the Community*.

[11] Ben Natan M, Ben Ari G, Bader T, Hallak M (2012). Universal screening for domestic violence in a department of obstetrics and gynaecology: a patient and carer perspective. *International Nursing Review*.

[12] Bradley F, Smith M, Long J, O’Dowd T (2002). Reported frequency of domestic violence: cross sectional survey of women attending general practice. *British Medical Journal*.

[13] Husso M, Virkki T, Notko M (2012). Making sense of domestic violence intervention in professional health care. *Health and Social Care in the Community*.

[14] Taft A, Broom DH, Legge D (2004). General practitioner management of intimate partner abuse and the whole family: qualitative study. *British Medical Journal*.

[15] Beynon CE, Gutmanis IA, Tutty LM, Wathen CN, MacMillan HL (2012). Why physicians and nurses ask (or don't) about partner violence: a qualitative analysis. *BMC Public Health*.

[16] Baird K, Salmon D, White P (2013). A five year follow-up study of the Bristol pregnancy domestic violence programme to promote routine enquiry. *Midwifery*.

[17] Stinson CK, Robinson R (2006). Intimate partner violence: continuing education for registered nurses. *Journal of Continuing Education in Nursing*.

[18] Robinson R (2010). Myths and stereotypes: how registered nurses screen for intimate partner violence. *Journal of Emergency Nursing*.

[19] Basu S, Ratcliffe G (2014). Developing a multidisciplinary approach within the ED towards domestic violence presentation. *Emergency Medicine Journal*.

[20] Feder G, Davies RA, Baird K (2011). Identification and Referral to Improve Safety (IRIS) of women experiencing domestic violence with a primary care training and support programme: a cluster randomised controlled trial. *The Lancet*.

[21] Papadakaki M, Petridou E, Kogevinas M, Lionis C (2013). Measuring the effectiveness of an intensive IPV training program offered to Greek general practice. *BMC Medical Education*.

[22] MacMillan HL, Wathen CN, Jamieson E (2009). Screening for intimate partner violence in health care settings: a randomized trial. *Journal of the American Medical Association*.

[23] Klevens J, Kee R, Trick W (2012). Effect of Screening for partner violence on women’s quality of life. *Journal of the American Medical Association*.

[24] Recommendations for the prevention of interpersonal and domestic violence. Recognise, protect and act. http://pre20090115.stm.fi/ka1210341082447/passthru.pdf.

[25] Paavilainen E, Flinck A (2013). National clinical nursing guideline for identifying and intervening in child maltreatment within the family in Finland. *Child Abuse Review*.

[26] Flinck A, Leppäkoski T, Paavilainen E Identification of and Intervention in Intimate Partner Violence and Multiprofessional Education.

[27] Paavilainen E (1998). *[Child maltreatment in the family]. Perheen toiminta ja yhteistyö perhettä hoitavan terveydenhoitajan kanssa [Ph.D. thesis]*.

[28] Flinck A (2006). *Intimate partner violence as experienced by women and men—from Bed of Roses to Crown of Thorns [Ph.D. thesis]*.

[29] Guidelines for treatment situations with patients who have encountered family violence. http://www.uta.fi/hes/stoppia/index.html.

[30] Heiskanen M, Ruuskanen E Tuhansien iskujen maa. Miesten kokema väkivalta Suomessa. http://www.heuni.fi.

[31] McCloskey L, Lichter E, Granz M (2005). Intimate partner violence and patient screening across medical specialities. *Academic Emergency Medicine*.

